# The Sickness Rate in Germany

**Published:** 1897-04

**Authors:** 


### The Sickness Rate in Germany.

If national health is national wealth, then individual wealth is the politically important unit of measure. This is emphasized in an interesting and "taking" way in the introduction to a hand-book that is published by the Imperial Department of Health of the German Government. "The health of man is a precious possession. Its loss causes harm, not only to the individual, but also to the community. The individual whose health is impaired suffers discomfort or pain or both. He loses his ability to work or to earn a living and impairs his enjoyment of the pleasures of life. He must incur expense in order to regain his health, and anxiety and poverty are the results, both to himself and to his family. By the decrease of its productive labor the community suffers loss in its industries and incurs expense in the support of the sick, and when, as often happens, the sick man is attacked with an infectious disease, he becomes a danger to his neighbors. The extent of the loss arising from the impairment of health can be estimated from the returns of the Working-Men's Sick Clubs of Germany. In 1891, out of a total membership of 6,500,000, more than 2,000,000 cases of illness occurred, each of which averaged seventeen days in duration. These clubs paid, in medical expenses, $22,-000,000. Since it may be assumed that among the remaining 44,000,000 of the German population, 24,000,000 of whom are old enough to work, the cases of illness were as numerous and as long-continued as among the members of these clubs, the expense caused by sickness in Germany, in 1891, is not placed too high at $120,000,000. The loss incurred by stoppage of wages is not included in this sum of the preservation and promotion of the health of mankind in the aim of hygiene. Among the tasks which it propose are the prevention, limitation and removal of sickness and disease and the preservation and prolongation of the ability to work, and of man's life in general."

